# Multiple roles of Pol epsilon in eukaryotic chromosome replication

**DOI:** 10.1042/BST20210082

**Published:** 2022-02-07

**Authors:** Milos A. Cvetkovic, Esther Ortega, Roberto Bellelli, Alessandro Costa

**Affiliations:** 1 Macromolecular Machines Laboratory, The Francis Crick Institute, London NW1 1AT, U.K.; 2 Department of Macromolecular Structures, Spanish National Centre for Biotechnology, 28049 Madrid, Spain; 3 Centre for Cancer Cell and Molecular Biology, Barts Cancer Institute, Queen Mary University of London, London EC1M 6BQ, U.K.

**Keywords:** chromatin, CMG, cryo-EM, Ctf18, Pol epsilon, replisome

## Abstract

Pol epsilon is a tetrameric assembly that plays distinct roles during eukaryotic chromosome replication. It catalyses leading strand DNA synthesis; yet this function is dispensable for viability. Its non-catalytic domains instead play an essential role in the assembly of the active replicative helicase and origin activation, while non-essential histone-fold subunits serve a critical function in parental histone redeposition onto newly synthesised DNA. Furthermore, Pol epsilon plays a structural role in linking the RFC–Ctf18 clamp loader to the replisome, supporting processive DNA synthesis, DNA damage response signalling as well as sister chromatid cohesion. In this minireview, we discuss recent biochemical and structural work that begins to explain various aspects of eukaryotic chromosome replication, with a focus on the multiple roles of Pol epsilon in this process.

## Introduction

In all replicating systems a hexameric, ring-shaped helicase is loaded around DNA and uses the energy derived from nucleotide hydrolysis to unwind the double helix, providing the single-stranded DNA template for the replicative polymerases [1]. A direct comparison between the bacterial and eukaryotic systems gives a sense of the higher degree of complexity involved with replicating chromosomes in eukaryotes. Below, we highlight how the leading strand polymerase Pol epsilon is involved in handling this complexity, by participating in several aspects of chromosome replication that are unique to eukaryotes.

In bacteria, the initiator DnaA wraps around the double helix and promotes its melting [2]. DnaA also engages the DnaC loader that in turn promotes the recruitment of the DnaB replicative helicase around single-stranded DNA, in a process that is aided by ATP hydrolysis by DnaB. The ATPase function of DnaC supports DnaB loading, as well as its activation [3–7]. As a result, as soon as DnaB entraps single-stranded DNA, it starts replication fork unwinding [1]. On the contrary, in eukaryotic cells, replicative helicase loading and DNA unwinding are temporally separated [8]. In G1 phase, the initiator, ORC (a DnaA homologue) binds and bends the DNA [9] to load a set of two helicases forming a catalytically inactive head-to-head double hexamer around duplex DNA [10–12], in a process that requires ATP hydrolysis by MCM [13,14]. Activation of DNA unwinding requires the recruitment of a set of firing factors, including Cdc45 and GINS that engage MCM to form the CMG holo-helicase, which both melts and unwinds origin DNA [15–18]. A key role in CMG formation is played by the leading-strand polymerase Pol epsilon, which mediates the recruitment of GINS onto the MCM to promote CMG formation [18–21]. The mechanism of origin activation is only one of many fundamental differences between bacterial and eukaryotic chromosome replication. A second feature that is found in eukaryotes (and most archaeal species), but lacks in bacteria, is the packaging of DNA in nucleosome arrays [22–24]. Nucleosomes must be uncoiled ahead of the replication fork for DNA unwinding to occur, and the evicted parental histones have to be redeposited onto the duplicated DNA, interspersed with newly synthesised histones [25,26]. A key role in the histone redistribution on the two daughter strands is played, again, by Pol epsilon. This function is totally distinct from origin activation and indeed requires different factors within the Pol epsilon protein assembly [27]. A third major difference between bacteria and eukaryotes is the number of dedicated replicative DNA polymerases to extend the leading and lagging strands. While bacteria employ the same DNA Pol III polymerase to synthesise both strands, eukaryotes use two distinct polymerases, with Pol delta discontinuously synthesising Okazaki fragments on the lagging strand and Pol epsilon synthesising the majority of the leading strand template [28–31]. A fourth difference entails the mechanism to achieve processive DNA synthesis. In all domains of life, this involves the deposition of a sliding clamp onto a primer-template junction by the clamp loader. However, while in bacteria one of the clamp loader subunits physically links together the two Pol III polymerases behind the DnaB helicase [32], evidence that the eukaryotic RFC clamp loader is replisome-incorporated still needs to be acquired, although physical contacts with Pol epsilon have been reported for an alternative clamp loader involved in sister chromatid cohesion establishment [33].

In this review article, we describe how recent structural work is shedding light on the disparate functions of Pol epsilon in origin activation, replication fork progression and epigenetic inheritance.

## Origin activation

**Figure 1.**
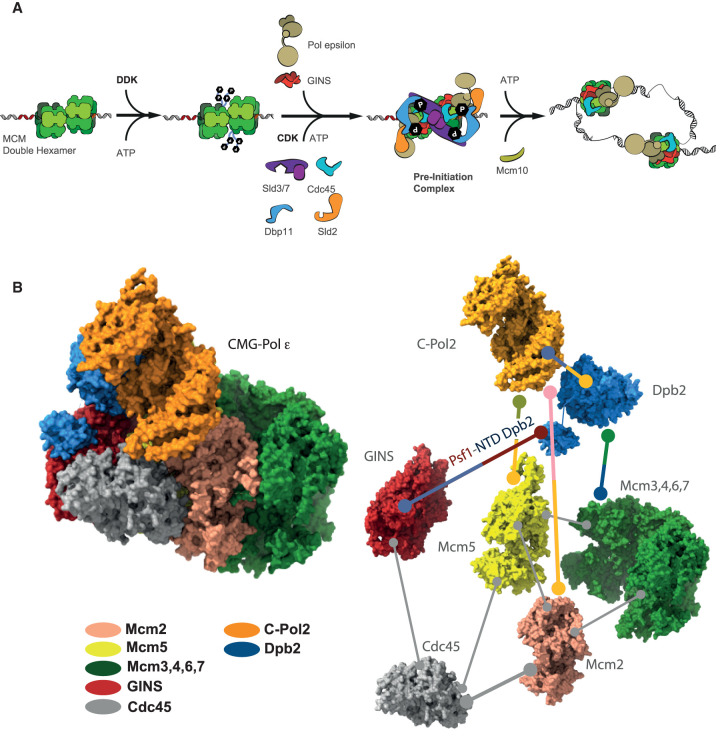
The role of Pol epsilon in replication origin activation. (**A**) Schematic representation of the cascade of molecular events leading to the replication fork establishment. (**B**) The structure of CMG bound to Pol epsilon explains the mechanism whereby Dpb2 and the Pol2 C-terminal domain recruit GINS to the MCM complex.

The first step towards the activation of origin-DNA-loaded MCM double hexamers is the phosphorylation of Mcm4 and Mcm6 subunits by the Dbf4-dependent kinase DDK [34–38]. The phosphorylated MCM subunits are recognised by the Sld3 phosphoreader [34], which exists in complex with the Sld7 dimer [39,40], yielding a dimer-of-dimers configuration. Sld3/7 are in turn responsible for the recruitment of the helicase activator, Cdc45 [39,41]. Recruitment of GINS, another helicase-activating factor, depends on a second kinase, CDK, which phosphorylates Sld2, promoting the formation of a so-called pre-loading complex that also contains Dpb11, GINS and Pol epsilon [20]. CDK has a second phosphorylation target amongst firing factors, Sld3. Phospho-Sld2 and phospho-Sld3 bind to a second phosphoreader, Dpb11, which contains a stretch of four BRCT, phospho-peptide binding repeats [42]. As a result of phosphorylation, a so-called pre-initiation super-complex is formed on origin DNA, containing MCM, Sld3/7/Cdc45 and GINS/Pol epsilon/Sld2/Dpb11 [18]. The pre-initiation complex is only loosely associated with the origin, according to *in vitro* studies. In fact, disassembly can be promoted upon high-salt treatment, which yields the loaded double hexamer scaffold. Tight association of a subset of factors can be achieved upon ATP binding by MCM, which leads to the retention of GINS and Cdc45 on the MCM, in turn causing disengagement of the double hexamer into two separate CMG particles and initial untwisting of duplex DNA. Mcm10 then promotes ejection of the lagging strand template from the MCM central channel, activation of ATP-hydrolysis dependent DNA unwinding and the recruitment of RPA that protects and stabilises the newly established replication fork (Figure 1A) [18].

Biochemical reconstitution experiments have demonstrated that omission of Pol epsilon prevents CMG formation and origin activation [21], while only a subset of functional domains are required in this process [43,44].

Pol epsilon is a tetrameric assembly composed of a catalytic subunit featuring a tandem repeat of polymerase domains. The first repeat is catalytically active, containing both DNA synthesis and exonuclease functions. The second repeat (C-terminal Pol2 or C-Pol2) has become inactivated during evolution, however, it is the only Pol2 domain essential for viability [45–48]. The second largest Pol epsilon subunit, Dpb2, contains an inactivated calcineurin-like exonuclease domain and is also essential for viability [49]. Finally, the dispensable Dpb3 and Dpb4 subunits contain a histone fold [50], which will be discussed in detail in the next section. C-Pol2 and the Dpb2 are required for CMG formation and DNA replication *in vitro*, explaining why these two elements are essential for viability [44]. Electron microscopy studies on the CMG and its interaction with Pol epsilon suggest a plausible structural basis for the essential role of C-Pol2/Dpb2 during origin activation. The first report of a CMG structure revealed a topologically closed MCM ring flanked by GINS and Cdc45 that contact the N-terminal domain of MCM and latch across a natural discontinuity in the helicase (Mcm2–5) [17]. Pol epsilon is positioned on the C-terminal, ATPase side of the MCM helicase [51] via its non-catalytic portion [52]. In particular, C-Pol2 contacts Mcm2 and Mcm5 via the catalytically defunct polymerase module and Dpb2 contacts Mcm3 via the inactive exonuclease. As a result, C-Pol2 and Dpb2 close the Mcm2–5 gate on the ATPase side of the helicase motor ring. Dpb2 also contacts the B domain of GINS subunit Psf1, via its N-terminal extension [44], explaining earlier observations that this interaction achieves the integration of Pol epsilon into the replisome and is required for initiation (Figure 1B) [19]. Altogether, the cryo-EM structure of the CMG-Pol epsilon complex invites a model whereby the two essential domains in Pol epsilon serve as a physical link between MCM and GINS, providing an explanation for the structural role of Pol epsilon in CMG formation [44]. It should be noted that the CMG-Pol epsilon assembly was reconstituted *in vitro* on a model DNA fork, by mixing overexpressed, pre-formed CMG with Pol epsilon in the presence of a nucleotide analogue. Whether the exact same interaction interfaces are recapitulated during origin-dependent CMG formation remains to be established.

## Chromatin replication

Once activated, the CMG helicase must uncoil nucleosomes ahead of the replication fork to achieve the unwinding of parental DNA, and at the same time re-package duplicated DNA filaments into nucleosome arrays [25,26]. Nucleosomes harbour an octameric protein core composed of a pair of four histones (H2A, H2B, H3 and H4), wrapped around a stretch of ∼147 base-pair DNA [53]. Post-translational modification of histones modulates gene expression, encoding an epigenetic programme that is inherited upon cell division. This process maintains cellular fitness and controls harmonious development in multicellular organisms [54].

**Figure 2.**
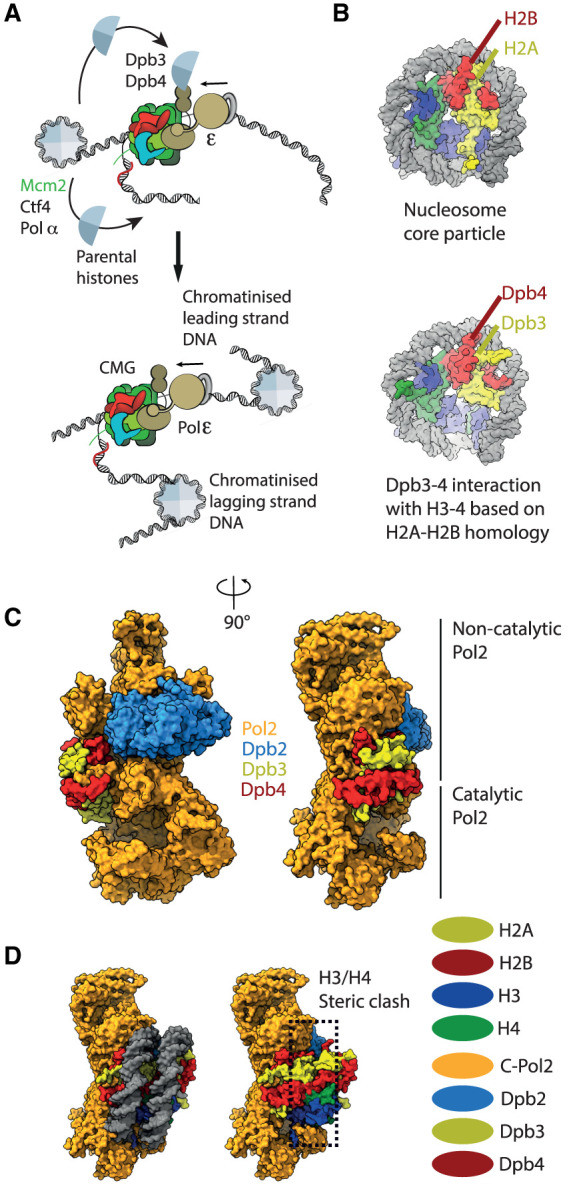
The role of Pol epsilon in chromatin replication. (**A**) Schematic representation of nucleosome uncoiling in front of the replication fork and parental histone redeposition onto duplicated DNA. (**B**) Modelled interactions between Dpb3–Dpb4 and histones H3–H4 based on the homology with histones H2A–H2B. (**C**) The structure of full-length Pol epsilon. (**D**) The rigid structure of full-length Pol epsilon would be able to accommodate an H3–H4 interaction with unstructured Dpb3–Dpb4 tails but not a histone-core-like engagement.

The mechanism of nucleosome uncoiling by the advancing replisome is not understood. Early work on CMG in the absence of DNA led to the suggestion that the helicase spools DNA through its central pore through a pumpjack-like movement of a set of C-terminal winged-helix domains appended to the MCM module [55]. Later structural work on the DNA-bound complex supports a model whereby the CMG, like other hexameric helicases [56–58], translocates along single-stranded DNA with a hand-over-hand, rotary-cycling mechanism. In this context, neighbouring ATPase sites in MCM ring subunits fire sequentially, causing conformational changes in DNA-interacting pore loops that promote nucleic acid rotation along the inner perimeter of the helicase channel, as well as translation from the N- to C-terminal side of the hexamer [59,60]. The CMG can bypass a roadblock on the lagging but not the leading strand [61,62], implying that the helicase employs a steric exclusion mechanism to split DNA at the fork nexus. A set of pore loops emanating from the N-terminal OB-fold domain of MCM handle the duplex:single-stranded DNA junction, with a conserved phenylalanine in Mcm7 forming pi–pi interactions with the pair base to be melted and a beta hairpin of Mcm3 diverting the lagging strand towards an exit passage formed between Mcm3 and Mcm5 [60,63,64]. Future work will elucidate how duplex-DNA opening is coordinated with nucleosome uncoiling ahead of the fork and which proteins within the replisome assembly facilitate this process. Several elements with histone chaperone function have been identified in core components of the replisome. Amongst these, a negatively charged N-terminal Mcm2 element can wrap around histones H3/H4 protecting a positively charged surface that would be left exposed upon DNA uncoiling [65,66]. An N-terminal domain in the catalytic DNA polymerase subunit of Pol alpha/primase is understood to serve similar functions [67,68]. The histone chaperone FACT (Facilitates Chromosome Transactions) has also been implicated as a replisome component [69–71], although it becomes essential in supporting replication through nucleosomes only when chromatin is densely packed [72,73]. While the role of replisomal histone chaperones in nucleosome uncoiling is unclear, it is established that these factors play a key role in the redeposition of parental histones onto duplicated DNA, which is fundamental for epigenetic inheritance [54]. Selective histone transfer from parental to lagging strand DNA depends on the histone chaperone domain located in the Mcm2 N-terminus, and is likely to occur at the front of the advancing CMG helicase, co-localised with Pol alpha, in turn linked to the CMG via the Ctf4 replisome-organisation hub [51,63,67,74–77]. Histone transfer onto the newly duplicated leading strand DNA depends on Pol epsilon and in particular the Dpb3–Dpb4 subunits [27] (in yeast, or Pole4–Pole3 in humans). *In vitro* reconstitution work demonstrated that human Pole3–Pole4 function as histone chaperones that can engage H3–H4, promoting tetrasome formation and DNA supercoiling [78] (Figure 2A). The structural homology with histones H2A–H2B [50] invites a tantalising model, whereby Pole3–Pole4 engage a histone H3–H4 tetramer, mimicking its interaction with the H2A–H2B (Figure 2B), and promoting histone redeposition onto the newly synthesised DNA, in a process understood to be supported by FACT [25,26,72]. The full-length structure of yeast Pol epsilon, however, reveals that Dpb3–Dpb4 dimer is clamped between the catalytic lobe and the non-catalytic half of the complex. This architecture confers rigidity to the whole structure [79] (Figure 2C), which is incompatible with a histone-core-like interaction with histones H3–H4 (Figure 2D). A role for Dpb3–Dpb4 (Pole4–Pole3) in the stabilisation of Pol epsilon complex is further supported by work in mice, demonstrating that Pole4 deletion destabilises the whole Pol epsilon complex, leading to embryonic lethality in inbred strains and developmental abnormalities and tumour predisposition in mixed backgrounds [80]. In agreement with the essential role of Pol epsilon in origin activation, *Pole4−/−* cells showed reduced origin activation and replicative damage. Structural flexibility in the Pol epsilon complex might be required for Dpb3–Dpb4 to engage parental histones. Indeed, several electron microscopy reports indicate that, in particular when engaged with the CMG, Pol epsilon exists primarily in a flexible state, with the catalytic domain of Pol2 free to move with respect to the rest of the complex [44,51,52,81,82]. Such flexibility might render Dpb3–Dpb4 free to engage the H3–H4 parental histones on the path to nucleosome reconstitution on the newly synthesised leading strand DNA by Pol2. Alternatively, Dpb3–Dpb4 engagement with H3–H4 might not involve the histone-like interface *per se*, but rather unstructured negatively charged tails of Dpb3 and Dpb4. *In vitro* interaction studies and hydrogen-deuterium exchange mass spectrometry analysis on human proteins support this notion [78]. Indeed, while POLE3-POLE4 and its yeast homologues can separately interact with H3–H4, the negatively charged C-terminal tail of POLE3 is required for the interaction of the POLE3–POLE4 subcomplex with H3–H4 in high salt concentrations [27,77]. Of note, this unstructured domain is predicted to emerge from the histone fold embedded in the core of Pol epsilon, suggesting an important role in histone recycling, likely in cooperation with FACT or other histone chaperones. Further structural analysis is needed to elucidate the mechanism whereby Dpb3–Dpb4 deposit parental H3–H4 onto the newly duplicated leading strand emerging from the N-terminal catalytic domain of Pol2. This work will be also essential to dissect the roles of Pol epsilon instability and defective histone redeposition upon loss of POLE3–POLE4 in mammalian cells [78,80].

## Structural dynamics in the eukaryotic replisome

**Figure 3.**
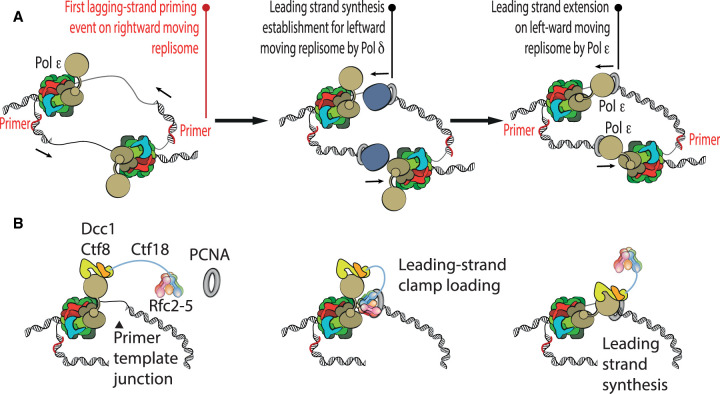
Mechanism of leading-strand priming and a role for Ctf18–RFC in establishing processive leading-strand synthesis. (**A**) Leading strand priming requires substrate hand-off from Pol delta to Pol epsilon. Priming of the leading strand on the leftward moving replisome occurs as the first lagging-strand priming event on the rightward moving replisome. (**B**) Ctf18–RFC constitutively binds Pol epsilon and loads the PCNA sliding clamp onto leading-strand DNA.

Establishment of DNA synthesis upon replication initiation requires the priming of both leading and lagging-strand templates by Pol alpha, with the primase subunit synthesising an RNA oligonucleotide, which is extended by the DNA polymerase subunit Pol1, before the substrate is handed over to processive replicative DNA polymerases [83]. The lagging-strand polymerase Pol delta plays a key role in the establishment of leading strand synthesis [43,84,85]. In fact, mapping of leading strand sites at origins of replication led to the discovery that leading strand synthesis occurring rightward from the origin is established by a lagging strand primer on the left of the origin and vice versa. The primer is extended by Pol delta, before substrate handoff to Pol epsilon. It is this substrate handoff that establishes continuous leading strand extension, differentiating it from discontinuous Okazaki fragment synthesis which will instead occur from the second lagging strand priming event onwards, as the two replisomes migrate in opposed directions [86] (Figure 3A). Whether and to what extent two diverging replisomes remain physically coupled is a matter of debate, with recent cryo-EM work proposing that two CMG helicases might remain associated via Ctf4 during DNA synthesis [76]. As Pol epsilon is bound to the MCM motor of the advancing replisome, structural flexibility is required to support substrate handoff from Pol delta to Pol epsilon. As introduced in the previous section, only the Dpb2 and C-Pol2 domains of the leading strand polymerase have been observed anchored to the CMG helicase, with the N-terminal catalytic domain of Pol2 flexibly tethered to the rest of the complex, in a configuration that would allow rapid substrate engagement (or disengagement, for example in response to DNA damage detection) [44,52]. Whether the CMG-associated Pol epsilon engaged in processive DNA synthesis adopts the rigid configuration recently described for the isolated apo polymerase assembly remains to be determined [79].

Single-molecule TIRF microscopy work on DNA replication reconstituted *in vitro* provides important knowledge on the compositional dynamics of the yeast replisome during replication fork advancement. For example, Pol delta was found to remain replisome-associated, supporting the synthesis of multiple Okazaki fragments, even when challenged with excess Pol delta in solution [87]. Similar observations have been obtained in single-molecule fluorescence microscopy work on yeast cells [88]. This replisome association depends on an interaction between the Pol32 subunit of the Pol delta and the Pol1 catalytic subunit of Pol alpha [87,89]. The observation that Pol delta remains engaged to the replisome was surprising and different from the commonly accepted notion that Pol delta synthesises Okazaki fragments by migrating away from the fork nexus. Pol epsilon on the other hand, remains stably associated with the CMG during leading strand extension, although it can be exchanged when challenged with excess Pol epsilon in solution [87]. This scenario is distinct from the substrate handoff between different polymerases, which would occur while the same Pol epsilon molecule remains anchored to the CMG [52]. Polymerase exchange has been observed in different replication systems [90–92] and can be explained with the postulation that several weak protein–protein interaction elements connect the polymerase with the rest of the replisome. In the CMG-Pol epsilon complex, these would be represented by the N-terminal domain of Dpb2 that links Pol epsilon to the GINS component of the CMG [19], and by a second interaction interface involving the C-Pol2/Dpb2 domains that contact the MCM ATPase [44]. We note that a change in the DNA engagement and nucleotide-binding state in the CMG can promote a large reconfiguration of the ATPase ring in the MCM [17,55,93], which would reconfigure the largest Pol epsilon interaction interface and could in turn promote polymerase ejection. The observed ability of the eukaryotic replisome to selectively exchange leading-strand polymerase might become critical to restart replication forks after stalling and could promote the recruitment of different polymerases required in the DNA-damage repair process.

## Achieving processive leading-strand synthesis

Biochemical observations on leading and lagging strand synthesis established that the isolated Pol epsilon is a more processive polymerase compared with Pol delta [94]. Crystallographic analysis of the N-terminal Pol2 catalytic domain explains this observation. Pol2 contains a ‘P-domain’ inserted in the polymerase fold, which is absent from Pol delta and achieves topological encirclement of the DNA substrate [95]. *In vitro* reconstitution demonstrated that interaction with PCNA (the sliding clamp that tethers the polymerase to the newly synthesised DNA) increases processivity of DNA synthesis both on the leading as well as on the lagging strand [43,94,96,97]. The structure of Pol delta bound to PCNA has been described by cryo-EM of human and yeast ternary complexes that also contain a primer-template junction DNA. Here, the C-terminal domain of the catalytic subunit is anchored to one of three PCNA protomers [98,99]. In this configuration newly synthesised DNA is threaded through PCNA, while other PCNA sites remain free to recruit the FEN1 nuclease for Okazaki fragment maturation [98]. How Pol epsilon contacts PCNA remains to be established [79]. PCNA is loaded onto DNA by the RFC clamp loader (a pentameric ATPase composed of Rfc1–5) [100]. Knowledge on any direct interaction between core replisome components and the clamp loader still needs to be acquired. Given that evidence for stable replisome incorporation of Pol delta has only been recently obtained [87], it is likely that studies on the architecture of the complete lagging-strand replisome might reveal specific interactions with RFC. Likewise, RFC can load PCNA for processive DNA synthesis by Pol epsilon on the leading strand, though direct protein–protein interactions with the leading strand replisome have not been described. However, a heptameric, alternative clamp loader where the Rfc1 subunit is swapped for Ctf18, can form a stable complex with Pol epsilon. Ctf18 exists in a complex with Ctf8 and Dcc1 [101–104], together forming a separate, hook-like module that engages the N-terminal Pol2 catalytic domain of Pol epsilon [33]. Notably, the heptameric Ctf18–Dcc1–Ctf8–RFC complex loads PCNA more efficiently than the pentameric complex missing Dcc1-Ctf8 [104,105]. Ctf18–RFC has been implicated in sister chromatid cohesion [106], checkpoint activation and DNA damage repair. A constitutive interaction between Ctf18–RFC and Pol epsilon suggests that this alternative clamp loader might be a core component of the leading-strand replisome. Although it is not required for viability [107], Ctf18–RFC is therefore well-positioned to protect stalled forks and promote S-phase checkpoint activation (Figure 3B) [33].

## Conclusions

Here we reviewed recent advances in biochemistry and structural biology that begin to explain the molecular mechanism of chromosome replication, with a focus on the multiple roles of Pol epsilon in different stages of origin activation and replication fork progression. To reach a complete understanding of the structural mechanism of DNA replication several questions need to be addressed. These include the mechanism of Pol epsilon incorporation into the pre-initiation complex, on the path to replication fork establishment; the mechanism whereby the Dpb3–Dpb4 subunits of Pol epsilon deposit parental histones H3–H4 onto leading strand DNA; a complete view of the leading-strand priming process and substrate handoff between Pol alpha, delta and epsilon, on route to processive and continuous leading strand synthesis; the mechanism whereby the clamp loader hands the clamp-engaged DNA substrate to Pol epsilon to achieve processive leading strand synthesis. Cryo-EM of reconstituted DNA replication reactions will play a major role in elucidating these processes.

## Perspectives

- Biochemical reconstitution and single-particle cryo-EM are shaping our understanding of DNA replication mechanisms.
- DNA unwinding and nucleosome uncoiling, processive DNA synthesis and the redeposition of parental histones are tightly coupled processes.
- Future cryo-EM on entire DNA replication reactions will provide a complete understanding of chromosome replication.

## Open Access Statement

Open access for this article was enabled by the participation of The Francis Crick Institute in an all-inclusive Read & Publish pilot with Portland Press and the Biochemical Society under a transformative agreement with JISC.

## Abbreviation

FACT: Facilitates Chromosome Transactions
